# Postsynaptic dorsal column pathway activation during spinal cord stimulation in patients with chronic pain

**DOI:** 10.3389/fnins.2023.1297814

**Published:** 2023-12-21

**Authors:** Gerrit Eduard Gmel, Rosana Santos Escapa, Teddy Edmond Benkohen, Dave Mugan, John Louis Parker, Stefano Palmisani

**Affiliations:** ^1^Saluda Medical, Macquarie Park, NSW, Australia; ^2^Guy’s and St. Thomas’ NHS Foundation Trust, London, United Kingdom

**Keywords:** spinal cord stimulation, neuroanatomy, electrophysiology, postsynaptic dorsal column pathway, neuropathic pain, mechanism of action, neural response recordings

## Abstract

Spinal cord stimulation (SCS) treatment for chronic pain relies on the activation of primary sensory fibres ascending to the brain in the dorsal columns. While the efficacy of SCS has been demonstrated, the precise mechanism of action and nature of the fibres activated by stimulation remain largely unexplored. Our investigation in humans with chronic neuropathic pain undergoing SCS therapy, found that post-synaptic dorsal column (PSDC) fibres can be activated synaptically by the primary afferents recruited by stimulation, and axonically by the stimulation pulses directly. Synaptic activation occurred in 9 of the 14 patients analysed and depended on the vertebral level of stimulation. A clear difference in conduction velocities between the primary afferents and the PSDC fibres were observed. Identification of PSDC fibre activation in humans emphasises the need for further investigation into the role they play in pain relief and the sensory response sensation (paraesthesia) experienced by patients undergoing SCS.

## 1 Introduction

Spinal cord stimulation (SCS) is a widely-used therapy for chronic pain management with a proven track record of efficacy and a favourable safety profile compared to pharmacological therapies (Kapural et al., 2015; North et al., 2016; Deer et al., 2018; Thomson et al., 2018; Mekhail et al., 2019; Russo et al., 2020). Although the exact mechanisms of action are not completely understood, it is established that stimulation of primary afferent (PA) mechanosensory fibres produces pain relief in patients with chronic neuropathic pain (Sdrulla et al., 2018). This principle is the basis for the development of a range of neuromodulation therapies targeting these fibres in different locations along their pathway, from the periphery (e.g., transcutaneous electrical nerve stimulation (Vance et al., 2014)), to dorsal root ganglion stimulation (Pope et al., 2013; Krames, 2015), and dorsal column stimulation (i.e., SCS).

The dorsal columns present an ideal target for the stimulation of primary sensory afferent fibres as they compose one of the major pathways conveying non-nociceptive sensory information. Recently, the development of closed-loop SCS, which maintains a constant level of dorsal column fibre activation by automatically adjusting the stimulus amplitude, has been shown to be superior to open-loop stimulation paradigms (paradigms with constant stimulus amplitude; Mekhail et al., 2019). To achieve closed-loop control, the Evoked Compound Action Potential (ECAP), representing the summation of all action potentials elicited by a given stimulus pulse, is measured. ECAPs obtained from dorsal column stimulation have been shown to include primary sensory afferent fibres conducting in the Aβ range, lending credence to the mechanism of action of SCS being mediated by activation of these fibres (Parker et al., 2012).

However, the anatomy of the dorsal columns is more complicated than typically taught. For example, although they are mainly known for their role in conveying cutaneous sensory information, they also contain proprioceptive fibres. Furthermore, they contain small fasciculi of descending fibres carrying information from adjacent vertebral levels (i.e., the fasciculus septomarginalis, fasciculus interfascicularis, and the Philippe-Gombault triangle; Schuenke et al., 2015). There is also evidence, from animal models, for postsynaptic fibres ascending in the dorsal columns conveying touch and visceral pain information (Angaut-Petit, 1975; Cliffer and Giesler, 1989; Al-Chaer et al., 1996; Willis et al., 1999; Abraira et al., 2017).

In this study, we set out to determine how various stimulus paradigms affect fibre recruitment and patient sensation and to investigate the components of neural activation from SCS in patients with chronic neuropathic pain along their implanted electrode arrays. Results demonstrating the effect of stimulation frequency on both fibre recruitment and patient sensation have been published elsewhere (Gmel et al., 2021). This text focuses on our efforts to determine if fibres other than primary afferent cutaneous sensory fibres are activated during stimulation.

## 2 Methods

### 2.1 Experiment setup

Twenty patients undergoing a SCS trial for chronic neuropathic pain in the lower back and/or lower limbs were recruited into the study. The study protocol received Ethical Committee approval (REC Reference: 18/LO/0344, April 2018) and informed consent was obtained from all patients.

All patients underwent an epidural SCS trial with two 8-contact leads with 8 mm inter-electrode spacing (Nevro, Redwood City, United States). The leads were inserted in the posterior epidural space according to standard practice, aiming for an overlap of 2–4 contacts around the T9/T10 intervertebral disc, which resulted in a span of three vertebral levels approximately between the two leads. Recordings were taken during two routine follow-ups by connecting the externalised leads to a custom external stimulator, capable of simultaneous real-time recording from each contact (Saluda Medical MCS Mk II), as described previously (Parker et al., 2013). Responses were filtered with a 4 kHz single-pole anti-aliasing filter and sampled at 30 kHz with a United Electronic Industries data acquisition system (Walpole, MA, United States) and a 10 Hz high-pass filter. The data acquisition unit itself contains an anti-aliasing filter at the Nyquist frequency (15 kHz). Neural recordings (ECAPs) were obtained from all electrodes not used for stimulation.

To limit the amplitude of stimulus artefact, we used one of the implanted electrodes as a reference channel for the bioamplifiers in patients 1–4 and 6. In effect, this creates a differential recording setup which distorts the ECAP signal observed by the amplifiers and makes a detailed analysis of the neural components of the signal difficult. For patients 5 and 7–20, an external pad electrode was used (Model No: 041826, Medtronic, Dublin, Ireland) as reference channel in order to achieve single-ended recording of the ECAP signals elicited by SCS. Patient 8 withdrew from the study after the first visit and ECAP threshold was never reached in that patient. Patients 1–4, 6 and 8 were therefore excluded from the analysis.

For each patient, stimulation was applied to various locations along the electrode array with an aim to stimulate at least at the top, middle, and bottom of the array (time and patient comfort permitting). In patients where more than one neural population was observed, additional stimulation locations were investigated to determine their anatomical occurrence as precisely as possible.

Stimulation settings were chosen to optimise the signal-to-noise ratio (SNR) and consisted of biphasic, tripolar pulses at either 30, 50, 100, 240 or 400 μs and 12, 20, 30 or 33 Hz. In these so-called “current sweeps,” the current was increased while maintaining all other variables constant until the stimulation sensation tolerable limit of the patient was reached, what is referred to as the “patient maximum.” Table 1 shows all experiments in which the SNR was good enough for reliable ECAP peak detection.

**Table 1:** Range of stimulation locations with analysable signal-to-noise ratios among the study cohort.

Patient ID	Lowest vertebral level	Highest vertebral level	Number of sweeps	Highest vertebral level stimulated where two fibre populations were elicited
5	Top T11	Top T8	7	Top T11
7	T11/T12 disc	Top T6	6	T10/T11 disc
9	T10/T11 disc	Top T8	4	T10/T11 disc
10	Top T11	Mid T8	9	None
11	T11/T12 disc	Mid T10	4	None
12	Top T11	Top T8	7	Top T11
13	Mid T11	Mid T8	7	None
14	Bottom T11	Bottom T8	3	None
15	Top T11	Top T11	1	Top T11
16	Bottom T11	Mid T8	5	Bottom T10
17	Mid T11	Mid T8	10	Mid T9
18	Bottom T10	Top T8	7	Bottom T10
19	Mid T11	Top T8	5	Mid T11
20	Mid T10	T7/T8 disc	4	None

The number of sweeps represents the number of current ramps (from 0 to patient maximum) that were carried out at various locations within the vertebral range. The highest vertebral level is that for which secondary ECAPs were observed orthodromically (note that secondary ECAPs were also observed when stimulating below that level but not above). All electrode locations were obtained from the x-rays of the final lead placement during the lead implantation surgery or additional x-rays obtained when lead migration was suspected.

### 2.2 Analysis

**Figure 1:**
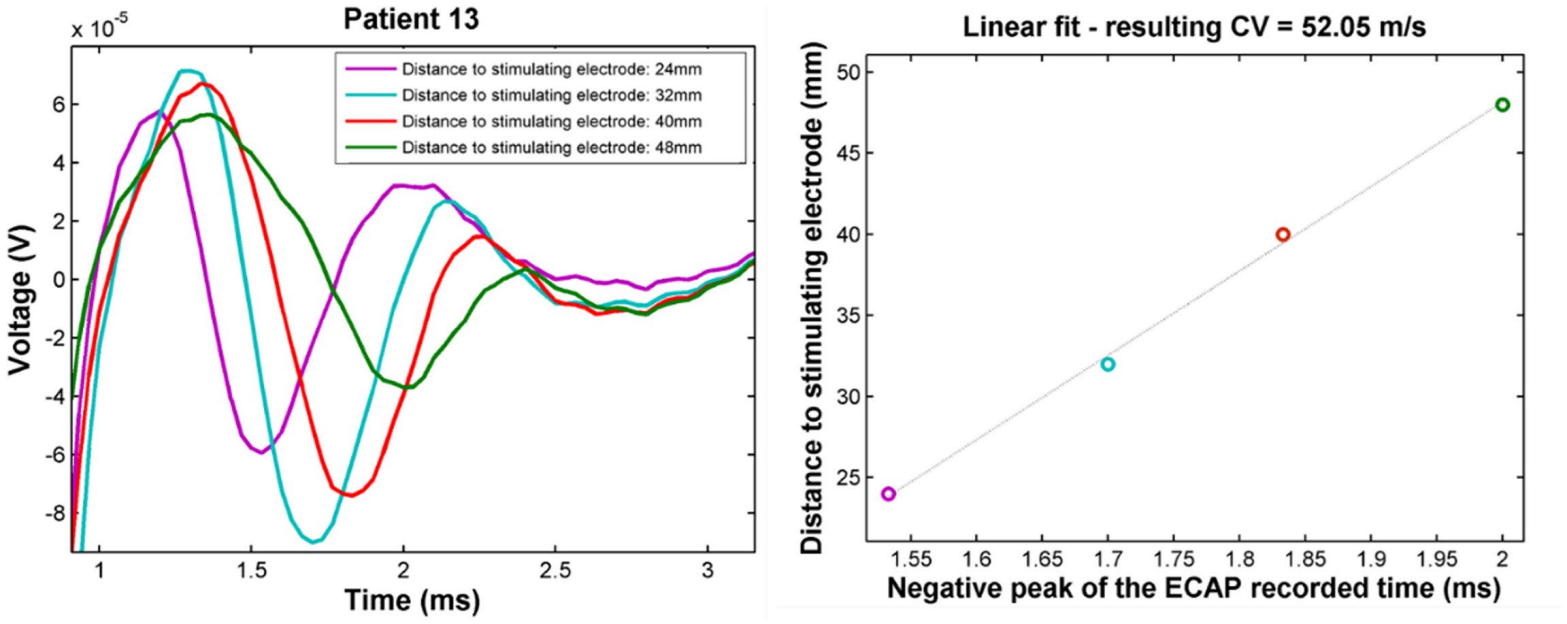
Example propagation plots for patient 13. Left: propagating ECAP observed for stimulation at T11 (stimulation pulse applied at time *t* = 748 μs). Right: propagation of the N1 peak latencies versus distance from stimulation. CV of the ECAP is taken as the inverse of the slope of the linear fit of these points. The CV in this case was found to be 52 m/s.

After the patient visit, the ECAPs were analysed at the patient maximum level (to maximise the SNR). All ECAPs obtained at this stimulation amplitude were averaged to reduce noise (the number varies between experiments but was always higher than 100). The negative peaks present in the averaged signal (which correspond to the largest peaks of 3-lobe ECAPs) were measured using custom MATLAB (v. 2012b) software and identified them in order of appearance as N1 and N2. On electrodes where the stimulus artefact was larger than the ECAP, an exponential was fitted and removed to approximate the artefact shape to facilitate peak detection. The location of each electrode in the spine was derived from the latest available x-ray and classified as either one of the top, middle, or bottom of each vertebra, or on the intervertebral disc. The peak latencies (time difference between the ECAP peaks and the end of the stimulus pulse) were compiled into propagation plots showing the antidromic (caudal to stimulation) and orthodromic (rostral to stimulation) propagation of the negative peaks of the neural signal (see Figure 1 for example). The conduction velocity (CV) of each peak was then obtained from the slope of a linear least-squares fit of the peak latencies. Two-sided t-tests were performed to determine the statistical significance of the observed differences in the mean values of the conduction velocities.

## 3 Results

### 3.1 Characterisation of neural responses

Fourteen patients were implanted with two 8-contact leads, with 2 to 4 contacts overlapping around the T9/T10 vertebral interspace, as previously described (Gmel et al., 2021). Seventy-nine current sweeps were carried out across this patient population at various vertebral locations spanning T6 to the T11/T12 intervertebral disc (Table 1).

**Figure 2:**
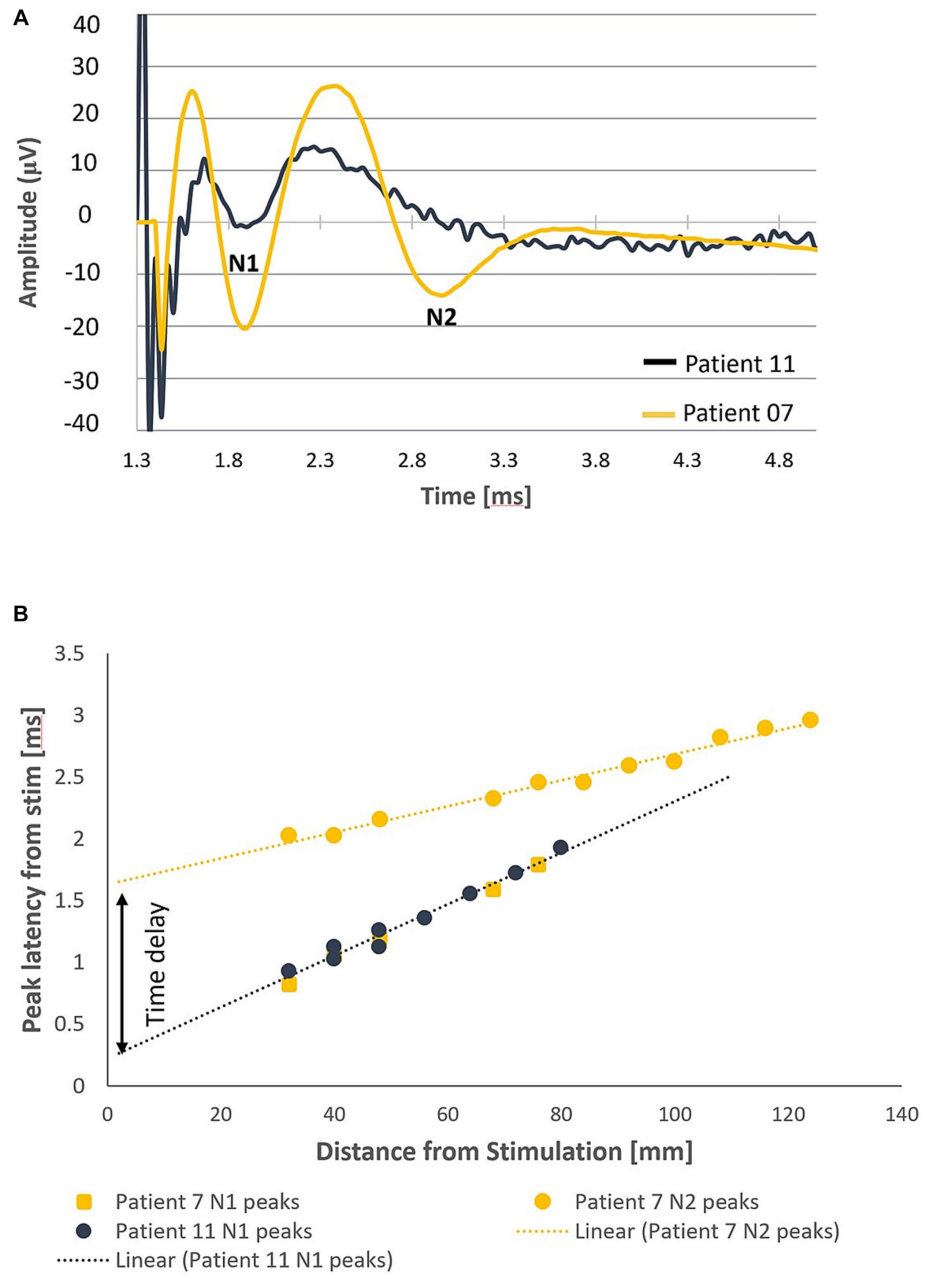
Orthodromic neural responses of Patients 07 and 11 when stimulating at T11/T12 intervertebral disc. **(A)** Elicitation of a primary ECAP alone in patient 11 and a secondary ECAP in patient 7 (stimulation pulse applied at time t = 958 μs). **(B)** Negative lobes of the primary and secondary ECAPs (labelled N1 and N2, respectively, normalised to the end of the stimulation pulse) propagate at distinct CVs away from the stimulus site (47.3 m/s and 94.8 m/s respectively). The secondary ECAP delay in initiation, measured as the difference of the y-intercepts of the linear fits of the N1 and N2 propagation plots, is approximately 1.46 milliseconds after the primary ECAP.

In all patients, a primary ECAP (measured by the N1 peak) was elicited and propagated both orthodromically and antidromically (when electrode configuration allowed for measurement in both directions). In addition to the primary ECAP, a secondary ECAP (measured by the N2 peak) was observed in 9/14 patients (64.3%). Examples of the recordings taken from 2 patients (Patients 07 and 11) with stimulation applied around the T11/T12 intervertebral disc are shown in Figure 2A. In patient 11, only a primary ECAP can be observed, whereas a secondary ECAP is also observed in patient 7. In this example, the secondary ECAP propagates significantly faster than the primary ECAP (94.8 m/s vs. 47.3 m/s) while starting with a delay of about 1.46 milliseconds after the primary ECAP (Figure 2B).

It is worth noting that, as an alternative explanation to a time delay, it can be conceived that the two ECAPs are elicited at the same time but at different locations. This alternative explanation can be ruled out by observing that the x-intercepts of the two trendlines are about 145 mm apart (meaning that the stimulation pulses would have to activate a bundle of fibres 14.5 cm away without activating anything else in-between).

**Figure 3:**
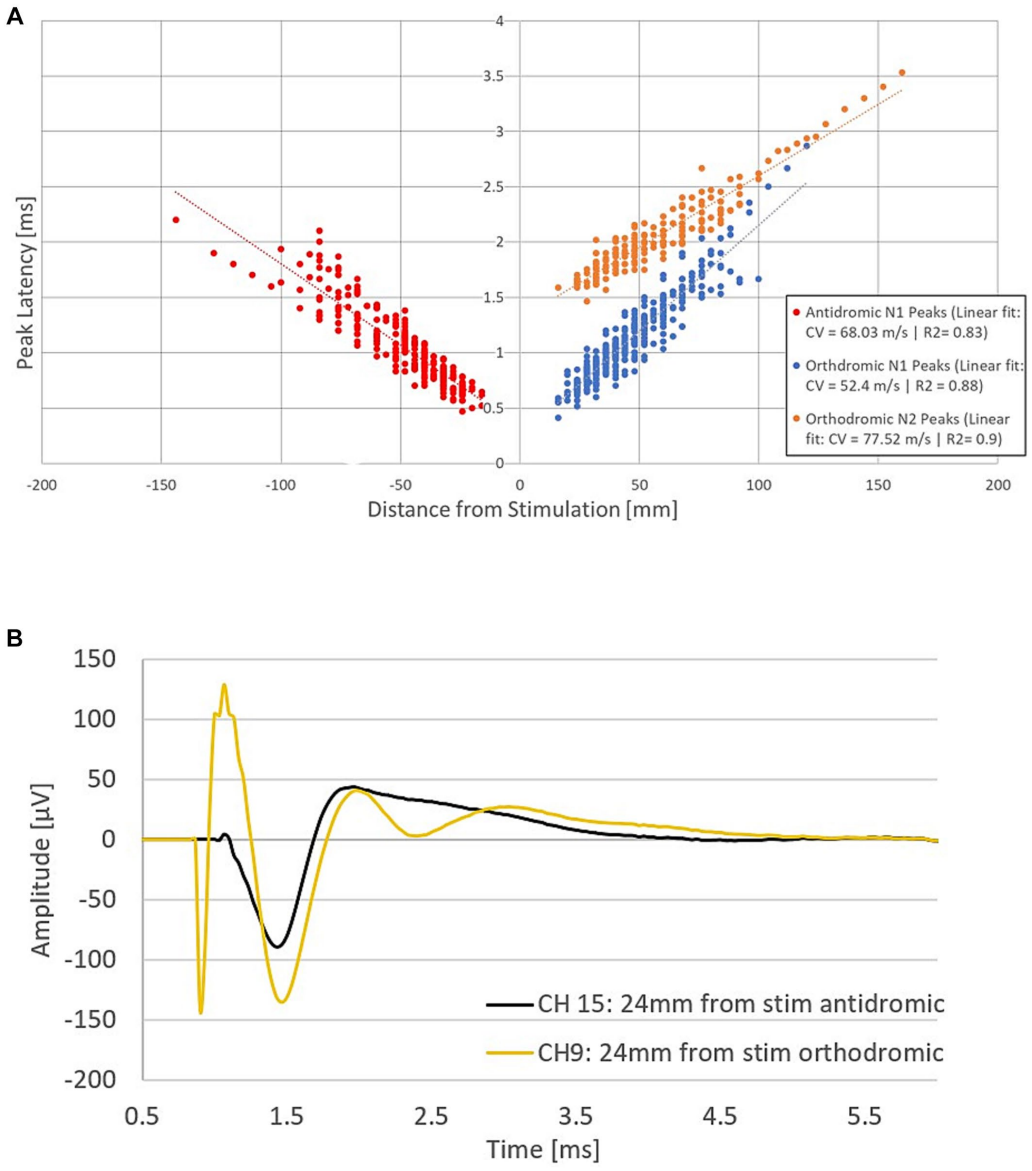
Fibre activation in patients undergoing spinal cord stimulation therapy for chronic neuropathic pain. **(A)** Propagation plot of 79 current sweeps conducted in fourteen patients. Delayed N2 peaks were observed in the orthodromic direction only. Note that by convention for this plot, negative values were attributed to distances in the antidromic direction. The antidromic N1 peaks originate from 13 patients, and 44 experiments. The orthodromic N1 peaks originate from 13 patients and 57 experiments, and the orthodromic N2 peaks from 9 patients and 23 experiments. Latencies normalised to the end of the stimulation pulse. **(B)** Representative example from patient 16 showing secondary fibre propagation only orthodromically. Stimulation on CH12 (located at the bottom of T10) at 40 mA, 30 Hz, 30us PW and recording at a distance of 24 mm from the stimulation in both the antidromic and orthodromic directions (stimulation pulse applied at time *t* = 748 μs). The secondary ECAP is only observed in the orthodromic direction.

**Figure 4:**
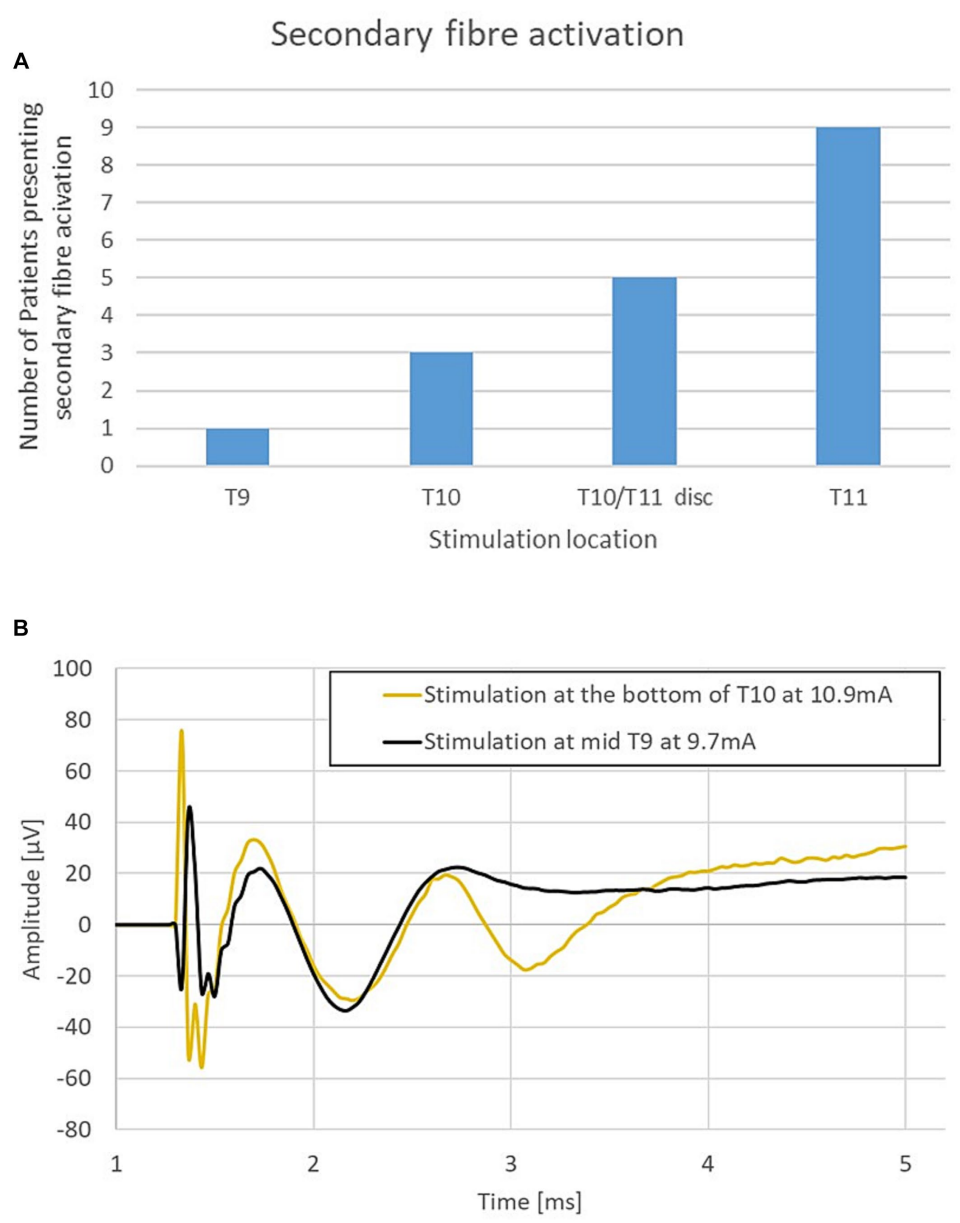
**(A)** Secondary fibre activation prevalence according to vertebral level stimulation. **(B)** Representative example from patient 18. Stimulation at T10 elicits a secondary ECAP while stimulation at mid-T9 does not. Stimulation done at 30 Hz, 240us PW (stimulation pulse applied at time *t* = 958 μs).

The combined propagation plot of the antidromic and orthodromic ECAPs (both primary and secondary) obtained from all 79 experiments included in the analysis are shown in Figure 3A. In all instances where the secondary ECAP is present, it is elicited with a delay from the primary ECAP, is only seen propagating orthodromically (illustrated by the absence of N2 peaks on the right-hand side of Figure 3A; an example from patient 12 is shown in Figure 3B), and is faster than the primary ECAP. The presence of secondary ECAP activation is patient and stimulation level-dependent (Figure 4A). An example from patient 18 is given in Figure 4B where stimulation at the bottom of T10 yields a secondary ECAP while stimulating at mid T9 does not. We observed that once there is a secondary fibre activation at a certain vertebral level, stimulation below this point will also elicit such activation (note that no testing stimulation was done below the T11/T12 intervertebral space owing to lead placement). Secondary fibre activation at T9 was observed in only one patient. In all other patients, secondary fibre activation was restricted to stimulation at the T10 or below (Figure 4A). It should be noted that there was no change in patient-reported quality of stimulation sensation, or sensation in general, when the secondary fibres were activated compared to when they were not.

### 3.2 Conduction velocities of neural response components

The average conduction velocity (CV) of the N1 and N2 peaks can be measured in 2 ways: Method 1, taking the slope of the linear fit of the propagation plot (Figure 3A); or Method 2, taking the slope of the linear fit for individual experiments (separating antidromic from orthodromic propagation) and computing the average of the obtained CVs. The results from both methods are shown in Table 2 along with the measure of the onset delay of the orthodromic N2 compared with the orthodromic N1 peaks. Note that for the second method, only those CV values derived from at least 3 data points were included (this reduced the number of experiments from 79 to 77 for this analysis). Using Method 2, the average N1 antidromic CV (primary ECAP) was found to be significantly different from the N1 orthodromic CV (mean difference of 6.87 m/s, *p* < 0.05). This could *a priori* indicate that CV is impacted by the propagation direction, or by the vertebral level of stimulation. Given the finite length of the lead array, antidromic measurements are more often made with stimulation on the higher vertebral levels (and vice versa). This question can be resolved by comparing the CV in either direction in experiments around the middle of the lead array (in which neural signals can be observed in both directions away from stimulation). We restricted our analysis to the subset of experiments in which at least 3 data points are present in each direction of propagation. Twenty experiments from 13 patients fit those criteria (stimulation location ranging from vertebral levels T9 to T10). In this subset, the N1 peaks propagate antidromically at 54.03 m/s and orthodromically at 52.34 m/s (mean difference of 1.69 m/s, *p* = 0.616). Thus, as one would expect, propagation direction does not impact CV in the dorsal columns.

**Table 2:** Conduction velocities and onset delay of secondary fibre response.

	N1 antidromic CV [m/s]	N1 orthodromic CV [m/s]	N2 orthodromic CV [m/s]	N2 onset delay [ms]
Linear fits of combined propagation plot	CV: 68.02 (*R*^2^: 0.83) *N*_exp_: 44 *N*_pat_: 13	CV: 52.35 (*R*^2^:0.88) *N*_exp_: 57 *N*_pat_: 13	CV: 77.52 (*R*^2^: 0.9) *N*_exp_: 23 *N*_pat_: 9	Delay: 1.07 *N*_exp_: 21 *N*_pat_: 8
Statistics on individual propagation plots (mean, SD, *N*)	Mean: 58.55 SD: 12.63 *N*_exp_: 42 *N*_pat_: 13	Mean: 51.68 SD: 9.15 *N*_exp_: 55 *N*_pat_: 13	Mean: 81.41 SD: 14.26 *N*_exp_: 22 *N*_pat_: 9	Mean: 1.12 SD: 0.15 *N*_exp_: 21 *N*_pat_: 8

Data derived from both the combined propagation plot and as summary statistics from the propagation plots of the individual experiments. *N*_exp_ denotes the number of experiments included in each group (one experiment can contain an antidromic and an orthodromic measurement), and *N*_pat_ the number of patients from which the measures are taken.

**Figure 5:**
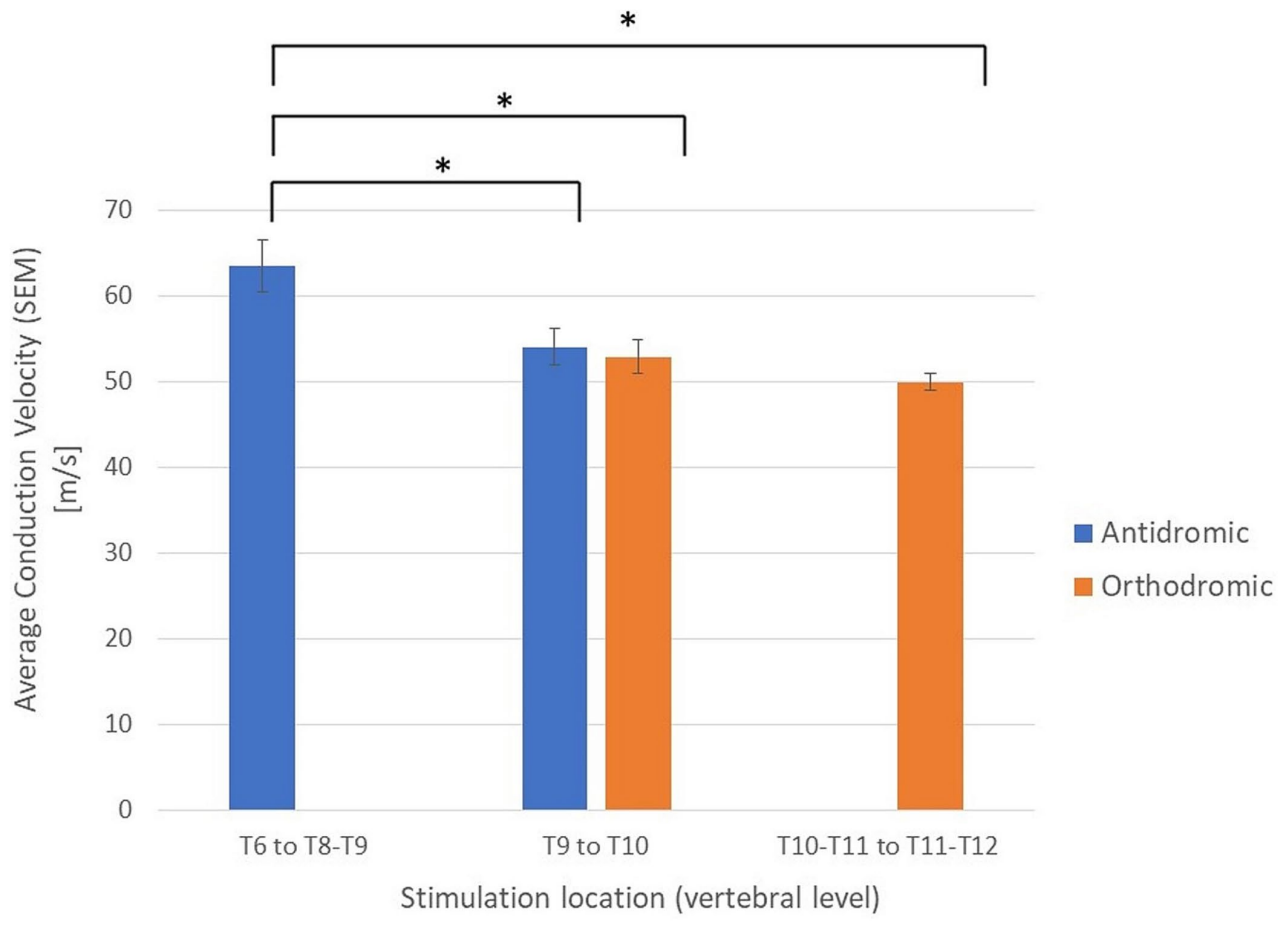
Average conduction velocities separated by propagation direction (orthodromic/antidromic) and by the vertebral level of stimulation. ^*^Student’s *t*-tests, *p* < 0.05; SEM, standard error of the means.

**Figure 6:**
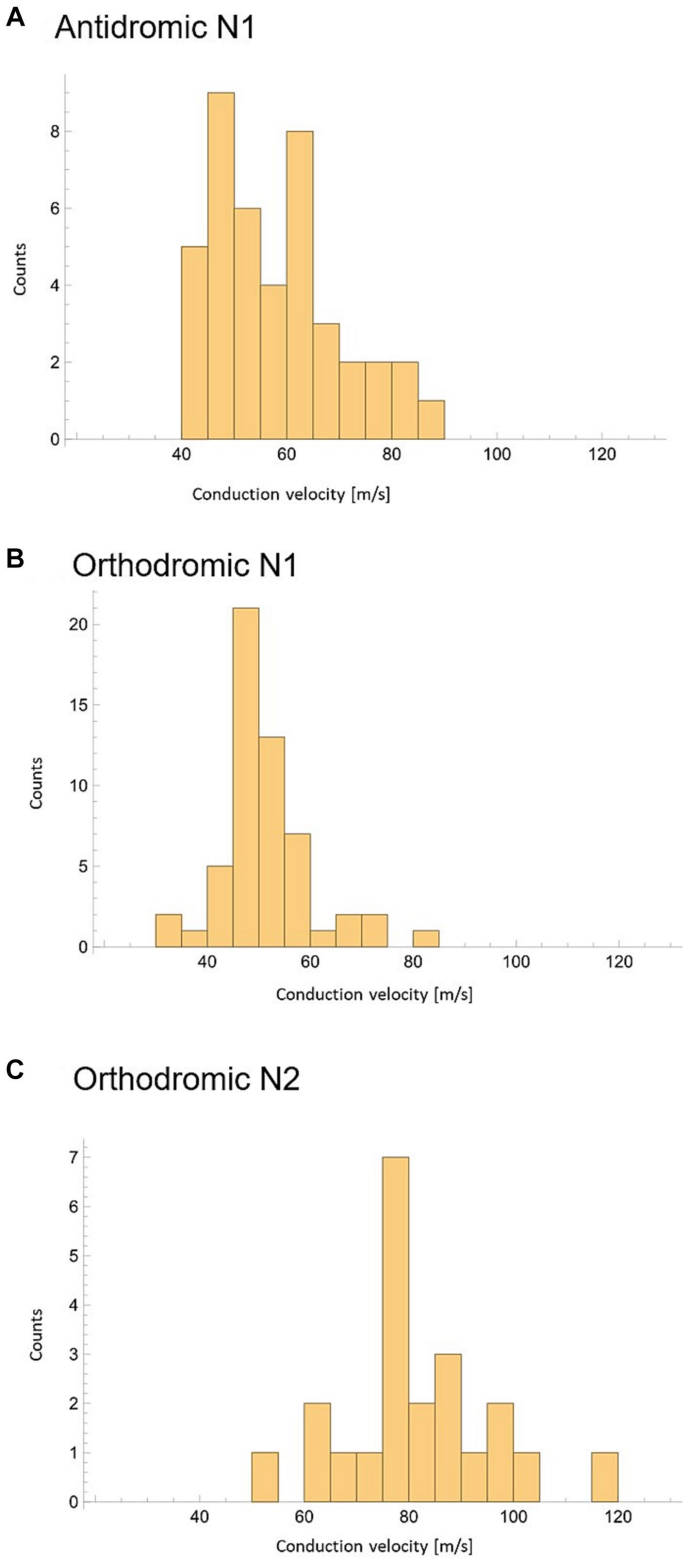
Peak conduction velocity recorded across all experiments with at least 3 datapoints available. **(A)** CV of N1 peaks propagating antidromically. **(B)** CV of N1 peaks propagating orthodromically. **(C)** CV of N2 peaks propagating orthodromically.

To investigate the possible effect of vertebral level on stimulation experiments were subdivided into 3 groups based on the vertebral level where the stimulation was applied: Group 1: Stimulation applied between the T11/T12 intervertebral space and the T10/T11 intervertebral space (23 experiments giving 23 orthodromic measurements); Group 2: Stimulation applied between T9 and T10 (34 experiments with a total of 54 CV measurements, 22 antidromic and 32 orthodromic measurements); and Group 3: Stimulation applied between T6 and the T8/T9 intervertebral space (20 experiments, giving 20 antidromic measurements). A significant increase in mean CV was observed when stimulation was applied at higher vertebral levels (Figure 5). It should be noted that the difference in CVs between Group 3 and Group 2 is significant, even when considering only the antidromic CV measures in each group. The difference between Groups 1 and 2 is not significant but trending in the same direction. However, it is clear that the observed difference in CV is linked to the vertebral level of stimulation and not simply the propagation direction. As useful as summary statistics are to paint a clearer picture, it is important to note that the CV measurements have shown a large degree of variability. For completeness, we have included histograms representing CV of the primary ECAPs (antidromically and orthodromically) and secondary ECAPs (Figures 6A–C).

## 4 Discussion

Through investigation of neural responses to electrical stimulation (ECAPs) at various lower thoracic vertebral levels in patients undergoing SCS therapy for chronic back and/or leg pain we have established that: (1) SCS in the lower- to mid-thoracic spine in patients with chronic pain elicits a secondary ECAP in over half the patients tested; (2) the presence of the secondary ECAP is more likely when stimulating at lower vertebral levels; (3) the secondary ECAP conduct at a significantly larger CV than the primary ECAP (approximately 80 m/s versus approximately 50 m/s); (4) both primary and secondary ECAPs have amplitudes of the same order of magnitude when measured on epidural electrodes; (5) when present, the secondary ECAP is elicited approximately 1 millisecond after the primary ECAP; and (6) there is a significant difference in CV of the N1 peak based on the vertebral location of the stimulation.

**Figure 7:**
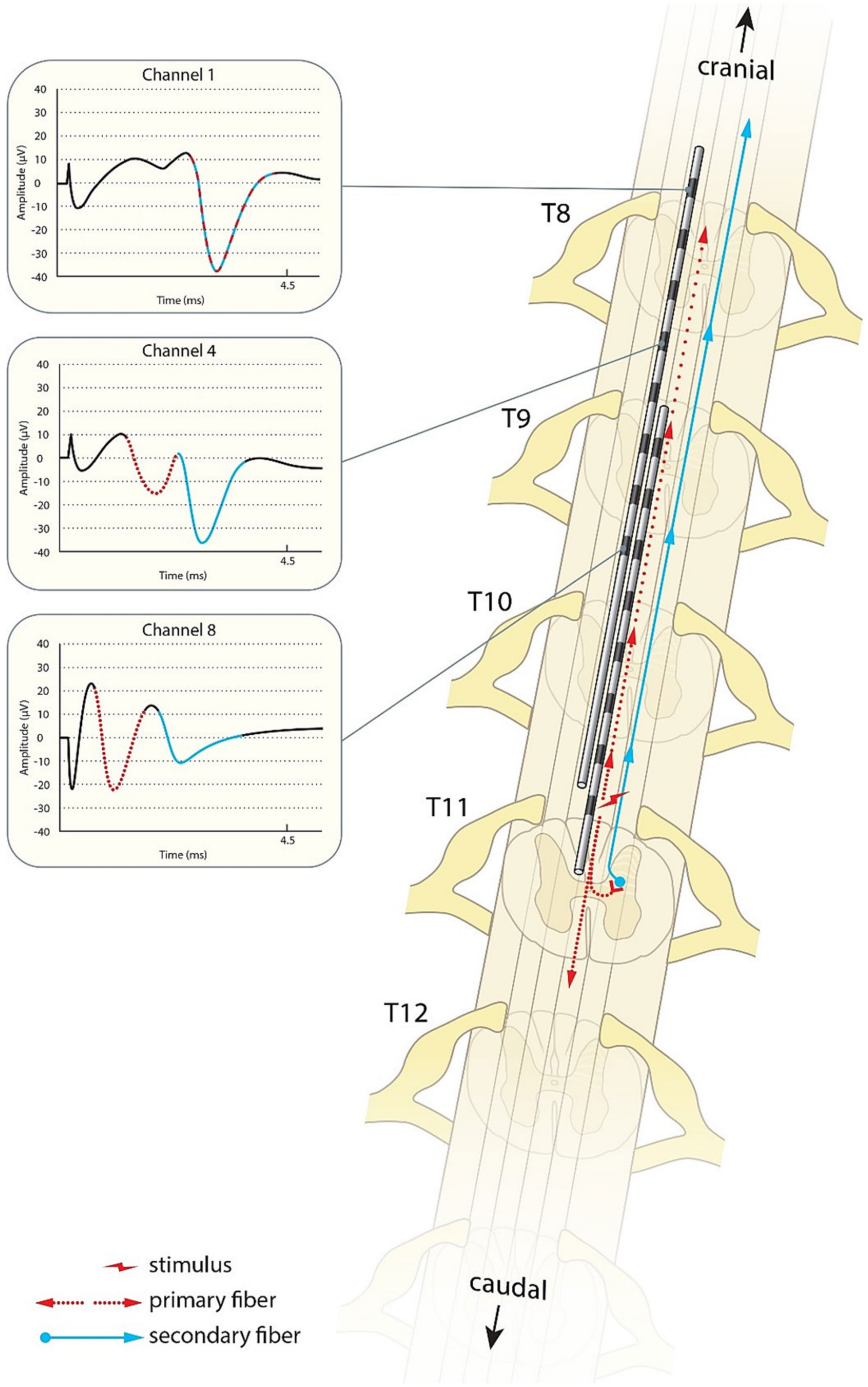
Schematic illustration of the primary sensory afferents (red) and postsynaptic dorsal column fibres (blue) activated by spinal cord stimulation. As shown in the traces from patient 17, the secondary ECAP is faster, but starts only after activation by the primary ECAP. The traces appear to “catch up” with each other as they ascend to the brain.

We postulate that these results best fit secondary fibres activated by the primary fibres whose nuclei are predominantly located in the lower thoracic spine, the secondary fibre axons then ascend to the dorsal columns within a segment or two where they then continue their ascent to the brain. Note that a *primary ECAP* can therefore consist of the combined action potentials of both primary and secondary fibres as long as the secondary fibres are activated directly by the stimulus pulse, rather than synaptically by the primary fibres. The difference in CV of the N1 peaks observed when stimulating at different levels along the spine are therefore likely representative of the ratio of primary fibres to secondary fibres. An N1 peak propagating at around 45 m/s would be almost exclusively made up of primary fibres, an N1 peak propagating at 90 m/s would be made up almost exclusively of secondary fibres, and peak CVs around 60–70 m/s are representative of a roughly even mix of primary and secondary fibres. A schematic representation of such a path is presented in Figure 7.

While the primary fibres are evidently the primary sensory afferents (PSA) which are known to be activated by SCS (Parker et al., 2012, 2013, 2017), the existence and nature of the secondary fibres is less well documented. A good candidate for the observed neural responses is the activation of the post-synaptic dorsal column pathway (PSDC) which, while having been described in the literature, specifically in the field of sensory processing, has been widely ignored in the field of pain management and spinal cord stimulation. As described in detail by Abraira and colleagues, primary sensory afferent fibres are processed in the dorsal horn laminae III and IV via monosynaptic and polysynaptic connections onto projection neurons which in turn project their axons via the dorsal columns to the brainstem dorsal column nuclei (Abraira et al., 2017). The existence of PSDC fibres terminating in the dorsal column nuclei has been established and studied widely in various animals and, while it has been shown that noxious stimuli can activate PSDC fibres, they are widely activated by innocuous stimuli and are likely to transmit mainly information of touch (Angaut-Petit, 1975; Bennett et al., 1983; Giesler et al., 1985; Kamogawa and Bennett, 1986; Cliffer and Giesler, 1989; Day et al., 2001). Interestingly, PSDC neurons have been shown to not participate in tactile allodynia as a result of spinal nerve ligation (Zhang et al., 2007). One shortcoming of our study was the lack of distinct qualitative descriptions of the stimulation sensation from each patient. We did not record any marked changes in the patients’ perception of the stimulation quality under stimulation paradigms which either would or would not activate secondary fibres. Further research should investigate whether patients can discern a change in sensation quality when secondary fibres are activated, this information could help identify the nature of the secondary ECAPs.

In recent years, another PSDC pathway has been identified as transmitting visceral pain (Willis et al., 1999; Houghton et al., 2001; Palecek et al., 2003; Krames and Foreman, 2007). It is unlikely that SCS would activate these fibres directly given the origin of their inputs and their nuclei situated in deeper layers of the dorsal horn.

As the data showed, the presence of a secondary response from stimulation depended both on the stimulation location on the cord (with lower stimulation locations more likely to elicit a secondary response) and the patient (a secondary response was observed only in 9/14 patients). The anatomical variability finds echoes in the literature, with PSDC neurons found predominantly in the lumbar and cervical enlargements in animals (Giesler et al., 1984; Enevoldson and Gordon, 1989).

This leaves the question of the inter-patient variability to which we unfortunately could not find clear answers. Given that PSDC neurons take inputs from a range of interneurons as well as the primary afferents, it is possible that the presence or absence of a PSDC response to stimulation could represent a sensitised or desensitised state of the synapse. This could either be representative of a pathological state, be linked to medication, or represent normal variability seen across a human population. Unfortunately, current medication intake was not recorded in this study. Short-term pain relief was not associated with the presence or absence of a PSDC response and information on the long-term pain relief likewise was not available for this study. These shortcomings will be addressed in future research by this group.

Sharma and colleagues studied synaptic activity generated at L1 by SCS applied at the T12/T13 levels in rodents (Sharma et al., 2023). Their group reported so-called evoked synaptic activity potentials (ESAPs) following the ECAP. Our recordings can unfortunately not be directly compared to theirs as we did not record in the lumbar region and they did not record in the orthodromic direction. Further, the ESAPs reported by Sharma and colleagues are elicited at 1 Hz, were not reported to propagate and were recorded from contacts much closer to the spinal cord in their preclinical preparation than our own. Dietz et al. (2022) also demonstrated propagating ECAPs in a rodent model at 4 Hz but did not report ESAPs, though stimulating elements, applied currents, and anaesthesia methods differed between the groups. Nevertheless, Sharma’s results are intriguing, in particular the observation of a reduction in ESAP amplitude after administration of an AMPA antagonist makes for a compelling argument as to the ESAP’s synaptic origin. Although ESAPs are distinct from the secondary ECAPs reported here, it is clear that SCS readily generates synaptic activity in proximity to the stimulation site. We hope that our work and that of Sharma and colleagues will be bridged in the future.

In light of the CV difference between the PA and secondary fibres, the changes in CV observed along the cord of the N1 peaks (often in the absence of an N2 peak) can likely be explained by the ratio of PA to secondary fibres. It is known that not all PSAs ascend to the dorsal column nucleus and that a majority of them terminate along the cord (Glees and Soler, 1951). The ratio of PSAs and PSDC fibres will therefore change along the cord which will be reflected in the CV of the observed ECAPs. This hypothesis finds support in animal work conducted by Idlett and colleagues who demonstrated that, in mice, dorsal column stimulation preferentially activates fibres which are distinct from primary afferents and conduct at a higher CV (43–69% faster than PAs; Idlett et al., 2019). Unfortunately, the group did not report orthodromic measurements that could be used to compare their work in mice with our ECAPs obtained from humans.

Despite being effective and widely used, the mechanism of action of SCS remains uncertain. To our knowledge, this is the first report demonstrating the activation of the sensory PSDC pathway in humans undergoing SCS for the treatment of chronic pain. Our group had, in the past, seen similar responses which were then termed “doublets.” These were however not investigated further at the time as they tended not to occur at stimulation locations used for therapy. Further research will continue to explore the role of anatomy, medication, anaesthesia, pain state, and stimulation sensation in the activation of the PSDC pathway by SCS. The findings highlight the need to revisit commonly accepted theories underpinning medical devices and open the door to improvements that are fact-based rather than rooted in trial-and-error. We hope that this article will spark further investigation into the mechanisms of action of spinal cord stimulation, elevate the field to a more evidence-based approach to therapy development, and bring advancements in ECAP measurement techniques to the attention of researchers in neuroscience.

## Data availability statement

The original contributions presented in the study are included in the article/supplementary materials, further inquiries can be directed to the corresponding author.

## Ethics statement

The studies involving humans were approved by Research Ethics Committee (United Kingdom) (reference: 18/LO/0344). The studies were conducted in accordance with the local legislation and institutional requirements. The participants provided their written informed consent to participate in this study.

## Author contributions

GG: Conceptualization, Formal analysis, Investigation, Methodology, Supervision, Writing - Original draft. RS: Data curation, Formal analysis, Investigation, Methodology, Writing – review & editing. TB: Data curation, Formal analysis, Investigation, Methodology, Software, Writing – review & editing. DM: Conceptualization, Funding acquisition, Project administration, Supervision, Writing – review & editing. JP: Conceptualization, Validation, Writing – review & editing. SP: Conceptualization, Investigation, Methodology, Supervision, Writing – review & editing.
